# Why did humans surpass all other primates? Are our brains so different? Part 1

**DOI:** 10.1590/1980-5764-DN-2024-0087P1

**Published:** 2024-04-15

**Authors:** Ricardo Nitrini

**Affiliations:** 1 Universidade de São Paulo, Faculdade de Medicina, São Paulo SP, Brazil.

**Keywords:** Primates, Brain, Language, Higher Nervous Activity, Syntax, Primatas, Encéfalo, Linguagem, Atividade Nervosa Superior, Sintaxe

## Abstract

This review is based on a conference presented in June 2023. Its main objective is to explain the cognitive differences between humans and non-human primates (NHPs) focusing on characteristics of their brains. It is based on the opinion of a clinical neurologist and does not intend to go beyond an overview of this complex topic. As language is the main characteristic differentiating humans from NHPs, this review is targeted at their brain networks related to language. NHPs have rudimentary forms of language, including primitive lexical/semantic signs. Humans have a much broader lexical/semantic repertory, but syntax is the most important characteristic, which is probably unique to *Homo sapiens*. Angular gyrus, Broca's area, temporopolar areas, and arcuate fascicle, are much more developed in humans. These differences may explain why NHPs did not develop a similar language to ours. Language had a profound influence on all other higher nervous activities.

## RESUMO

Esta revisão baseia-se numa conferência apresentada em junho de 2023. O seu principal objetivo é explicar as diferenças cognitivas entre humanos e primatas não humanos (PNH) com base nas características dos seus cérebros. Baseia se na opinião de um neurologista clínico e não pretende ir além de uma visão geral deste tema complexo. Como a linguagem é a principal característica que diferencia os humanos dos PNH, esta revisão concentra-se nas redes cerebrais relacionadas à linguagem. Os PNH têm formas rudimentares de linguagem, incluindo sinais lexicais/semânticos primitivos. Os humanos têm um repertório léxico/semântico muito mais amplo, mas a sintaxe é a característica mais importante e única do *Homo sapiens*. O giro angular, a área de Broca, as áreas temporopolares e o fascículo arqueado são muito mais desenvolvidos em humanos. Essas diferenças podem explicar por que os PNH não desenvolveram uma linguagem semelhante à nossa. A linguagem teve influência profunda em todas as outras atividades nervosas superiores.

## INTRODUCTION

### Higher nervous activities

This article was extracted from a conference presented at the Congress on Brain, Behavior and Emotions, held in Florianópolis, Brazil, on June 9, 2023. Professor José Eymard Pittella presented his conference entitled "The singularity of the human brain", based on his book "*O Cérebro que nos faz Humanos*" (The Brain that makes us Humans)^1^ and I presented "The development of higher nervous activities".

Prof. Pittela focused his presentation on anatomical differences among neurons, cortical areas, and connections,^2^ while my main objective was to explain the cognitive and behavioral differences between humans and other animals, based on physiological differences between brains. This review is based on the opinion of a clinical neurologist and does not intend to delve beyond an overview of this complex topic.

From the beginning, it is necessary to try to establish which are the cognitive or behavioral features that are most characteristic of a human being. Humans are social beings, but insects like ants, bees, and termites live in well-organized societies. Other primates also live in groups, although in smaller groups than humans.

The ability to create instruments was long considered a milestone, an essentially human trait. A study carried out in the Serra da Capivara, in Brazil, demonstrated that wild New World monkeys, the capuchin monkeys (*Sapajus libidinosus*), chip stones to use as tools to break very hard fruits and extract the nuts from the inside.^3^ The conclusion of that study was "that the production of archeologically identifiable flakes and cores, as currently defined, is no longer unique to the human lineage."^3^ The ability to produce tools was recently recognized in gorillas.^4^

Another highly developed skill in humans is the theory of mind or the ability to recognize what another individual is thinking or feeling.^5^ The grooming habit of monkeys suggests that they may have this ability. There are many cortical and subcortical networks involved in the complex ability to theorize about another's intentions, but domestic dog owners ensure that their dogs behave differently depending on their owners' moods, demonstrating that they can recognize feelings. However, there are still controversies about the existence of the theory of mind in non-humans, particularly because among its components in humans, language is very important.^6^ But the interpretation of feelings and emotions of others may depend less on language, and circuits involving the amygdala are known to be important in the development of the theory of mind.^6^ Therefore, theory of mind can be assumed to exist in animals, but in a much less complete form before the development of language.

Of all the activities that can be called higher nervous activities, language has been considered the one that most distinguishes us from other animals.

However, it must be recognized that animals do have some form of language. The already classic studies with vervet monkeys (*Chlorocebus pygerythrus*) from Africa, have shown that these primates emit different sounds when they perceive the presence of a leopard, eagle, or snake.^7,8^ When they hear the sounds emitted by members of the group, the other monkeys take measures for appropriate defense such as climbing trees, hiding in holes, or paying attention to the snake's movements, respectively. These sounds were recorded by researchers and when reproduced, the animals behaved according to the researchers' expectations.^8^ They also verified that very young monkeys emitted these sounds when they saw any mammal, a bird, or a stick, but as they grew old they learned to emit the appropriate sound in the right event to alert the group. In other words, they learned from the more experienced members of the group.^8^

The capuchin monkeys mentioned above emit 25 different types of vocalizations such as in situations of contact, alarm, aggression, sexual contact, during feeding, and whistles for long distance contact, for example.^9^

It is necessary to recognize that the differences between human beings and other mammals, especially other primates, are not radical but evolutionary differences.

**Figure 1:**
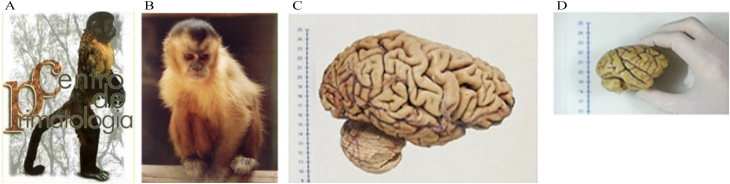
A) Primatology Center of the University of Brasilia; B) Capuchin monkey (*Sapajus libidinosus*); C) Human brain; D) *Sapajus libidinosus* brain (courtesy of Maria Clotilde H. Tavares and Roberta Diehl-Rodriguez).

The capuchin monkeys that we have studied in collaboration with the Primatology Center of the University of Brasilia, coordinated by Prof. Maria Clotilde H. Tavares (Figures 1A, 1, C) has allowed us to verify that the brain of capuchin monkeys has well-developed gyri (Figure 1D).

### The brain and the development of human language

**Figure 2:**
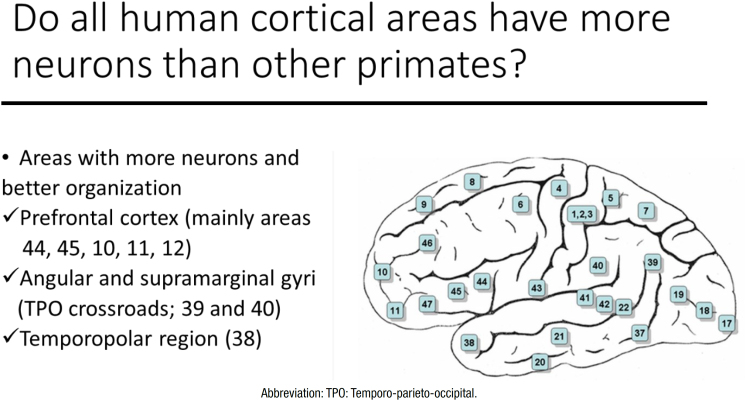
Brodmann's areas in the lateral brain surface.

The prefrontal cortex (PFC) is one of the most developed regions of the human brain when compared to other mammals.^2^ We will return to this subject when presenting the development of language in part 2. Within this large region of the dominant hemisphere (usually the left hemisphere, even for most of the left-handed) is Broca's area (Brodmann's areas 44 and 45) (Figure 2). Lesions of Broca's area cause intense difficulty in verbal expression.^10-12^

Most of our knowledge about the importance of a brain region for an activity was acquired from case reports, from studies of patients who had lesions in their brains. In 1841, when studying the clinical features of a patient who lost his ability to speak after a stroke and through the neuropathological examination, Pierre Paul Broca described a lesion in a region corresponding to areas 44 and 45 of the left hemisphere.^10-12^ A few years later, Karl Wernicke described that a lesion in the posterior part of the left superior temporal gyrus was associated with deep impairment of the understanding of the meaning of words.^10-12^

The idea of one center for each complex activity, such as language, caused a great discussion between the "globalists", on one side, who considered that the complex activities could not be linked to small centers in the brain, and the so-called "reductionists", who thought the brain may act as a mosaic of centers, each one being responsible for each activity.^10,11^

This discussion reached the end by the concepts of functional complete systems (developed by the Russian neuroscientists Anokhin and Luria)^10^ and cognitive networks (developed by Norman Geschwind), which are very similar. According to this framework, every complex and even simple functions are dependent on several connected areas of the brain, each making a particular contribution for the complete function.^10-12^ Maybe the first cognitive network that is still accepted was defined by Hugo K. Liepmann when studying apraxia at the beginning of the 20^th^ century, as described by Benson and Geschwind.^13^

According to this idea, there are several "systems" or networks inside the central nervous system. Currently, all these systems or cognitive networks are being discovered through neuroimaging or other techniques, which can detect areas with synchronous activity. The search for knowledge of the entire human "connectome" is one of the main research fields in today's neuroscience.^14^

The development of Broca's area and its connections with the motor areas allow us to speak about 10 to 15 vowels and consonants per second making less than one error per 1,000 words, which is the most complex cognitive-motor activity of the human being.^15^ Although this capacity has been very important for language development, it was admitted that the great development in the human brain of areas 39 and 40 was the most important for language development.^16,17^, These two areas are located at the temporo-parieto-occipital crossroads of the dominant hemisphere, which allow the capacity of representation or symbolization, in which a stimulus of one modality can be represented by another modality.^16,17^ Thus, pre-processed stimuli in the respective visual, auditory, or somesthetic unimodal association areas act on neurons located in the multimodal areas, such as the 39 and 40, allowing the cross-modality association. The possibility of representing one sensory modality by another, such as a visual perception by a sound (a name) and vice versa, or a sound by a gesture and vice versa, was and still is admitted as one of the main factors, which is responsible for the development of language.^16,17^

**Figure 3:**
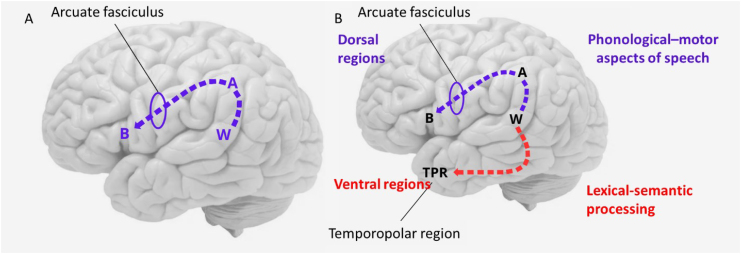
A) Wernicke-Lichtheim-Geschwind model of the cerebral organization of language; B) Phonological-motor aspects of speech: dorsal regions of the brain, including arcuate fasciculus and Broca's area; Lexical-semantic processing: ventral regions of the brain, including inferior longitudinal fasciculus and temporopolar regions. (Highly simplified schemas).

Norman Geschwind included the important participation of the angular gyrus (area 39) in the Wernicke-Lichtheim model of the cerebral organization of language, which since then has been known as the Wernicke-Lichtheim-Geschwind model.^16-20^ According to this, when the individual hears an articulated sound, the understanding of its meaning depends on the connection of the auditory association areas with Wernicke's area and with the angular gyrus, whereas, repetition of the articulated sound depends on the connections of Wernicke's area with Broca's area. The connection to Broca's area occurs mainly through the arcuate fascicle (or arcuate fasciculus), a highly myelinated bundle of axons that connects posterior areas of the auditory association cortex (and angular gyrus in the parietal cortex) with Broca's area (Figure 3A). The arcuate fascicle probably was one of the first tracts (or fascicles) of cerebral white matter to recognize function and importance.

We currently know that several fascicles located in the white matter of the cerebral hemispheres connect distant brain regions with each other and establish networks of connections that are responsible for the behavior and functionality of humans and other vertebrates.^21^

**Figure 4:**
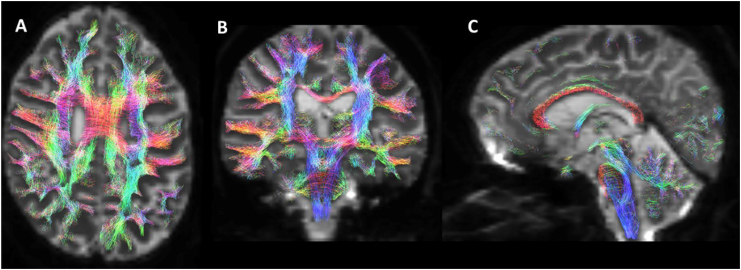
Post-processed diffusion magnetic resonance imaging images (MRtrix3 software) demonstrating association, projection, and commissural fascicles of the central nervous system. Views: A) axial; B) coronal; and C) sagittals. Courtesy of Diogo Fragoso Cardoso.

These fascicles allow for fast and accurate connections between different areas that constitute a complete functional system^22^ or cognitive network^16,23^ that are the basis for the manifestation of a complex behavior or function (Figure 4). Short fascicles, called U-shaped fibers, connect neurons located in neighboring gyri.^21^

### White matter matters

The white matter of the brain, which has this color mainly due to the myelin that surrounds the axons that constitute the fascicles, has always been very difficult for neuroanatomists to study. Advances in neuroimaging methods, especially diffusion tensor tractography, have allowed better identification of the fascicles involvement in different neuronal networks,^21^ always in conjunction with prior knowledge of neuroanatomy. The fascicles are identified because they reduce the free movement of molecules of water and thus increase the fractional anisotropy, which is a magnetic resonance imaging (MRI) index of white-matter organization that reflects improved fiber alignment and myelination.^21^

The identification of cortical and subcortical areas that are connected can also be performed by other neuroimaging methods or studies of brain electrical activity. Currently, functional MRI (fMRI) has been the most used. This method is based on selecting a region of interest (where a "seed" is placed) and verifying which regions are in sync with it, then forming a network.^14^

### Why are other primates not able to repeat the sounds and words we speak?

Non-human primates (NHPs) can imitate many human gestures, but they cannot repeat the sounds of the words we speak. This difficulty may depend on the lesser development of the area corresponding to Broca's area. However, a relatively recent study of diffusion tensor tractography that compared the arcuate fascicle of chimpanzees with that of humans demonstrated that in humans, the arcuate fascicle extends much more into the posterior temporal lobe,^24^ where the auditory unimodal association cortex is located.^23^ This, together with the lesser development of the area corresponding to Broca's may explain why NHPs do not repeat the sounds we emit. Hypotheses to explain the difficulty in repeating phonemes or words by NHPs have ranged from articulation difficulties due to differences between our oral-phonatory systems to difficulties in auditory identification of phonemes emitted by others. Between these two poles, some hypotheses highlight difficulties in the transmission of impulses from auditory areas and to the area corresponding to Broca's, which in turn, has insufficient development in NHPs. Besides, there are differences in the transferring of impulses between pre-motor areas and the motor area that innervate in a coordinated way, in humans, the muscles of the oral-phonatory system.

Newborn humans also cannot repeat what they hear, but with training, they acquire this ability. A study carried out by Perani et al. verified that diffusion tensor tractography of newborn humans does not identify the arcuate fascicle.^25^ To be visualized by tractography, it is necessary that the axons that form the fascicle are aligned and myelinated. In the absence of myelination, nerve conduction is very poor in the axons. With myelination of the axons of the arcuate fascicle over the first few months of life, nerve conduction is improved, and the ability of repeating develops progressively. Of course, this is associated with many other factors such as improved attention. It is curious to observe that the superior longitudinal fascicle that connects the posterior areas of the brain, including the auditory unimodal cortex with the frontal premotor areas (but not with Broca's area) was already well visualized by tractography in newborns in the study by Perani et al.,^25^ which indicates that this fascicle is already more organized and myelinated at birth. This may be related to the earlier development of lallation, which is a coarse form of imitation that precedes repetition.

Nonetheless, the ability to repeat words is not a uniquely human trait, because a few birds can repeat words and short phrases.

### The brain dictionary

Vervet monkeys emit alarm sounds with different meanings, and capuchin monkeys emit at least 25 context-related vocalizations, but humans have a huge dictionary in their brains with 20,000 to 35,000 words, according to an English report.^26^

This may be the essential difference between humans and other primates, for example.

Over time, neurologists and speech pathologists found that the difficulty in naming objects occurred when strokes, tumors, or other types of injury affected the cortex of the left cerebral hemisphere, and more frequently, when located in the temporal lobe.^27^

In 2016, Huth et al. investigated with fMRI the brain regions that were stimulated when normal volunteers listened to words presented in a text; and they reached a few surprising conclusions.^28^ The first is that very large areas of our cortex brain are stimulated when we hear words and that the cortex is almost "tiled by words".^28^ They also verified that both hemispheres are stimulated.

There was considerable overlap in the findings among survey participants but the most important conclusion of the article was that words are semantically organized in the human cerebral cortex.^28^ Thus, words like mother, wife, son, brother, and pregnant stimulate small areas very close to each other. They confirmed what we already knew, based on studies of patients with brain injuries, that words that identify colors, numbers, or body parts are organized semantically because there can be almost specific difficulty in naming these items in lesions of specific cortical areas. They also found that each word stimulates a very small area of the cerebral cortex, but that the same word does not stimulate just a small area. The word "top", for example, can stimulate areas close to those stimulated by numbers, buildings, or clothes, according to its different meanings.^28^

### The temporopolar region and the brain dictionary

In the classic model of human language organization, based on clinicopathological observations, speech understanding depended on the Wernicke's area located in the posterior portion of the left superior temporal gyrus, which includes the posterior part of the auditory association cortex,^12^ (although Wernicke's area was never well-defined from an anatomical point of view).^29^

In the most current model of human language, which was developed mainly from the studies of primary progressive aphasia (PPA), the meaning of words, or semantic knowledge, is closely linked to the temporopolar region of the left cerebral hemisphere. This region, which includes Brodmann's area 38, is considered critical for understanding the meaning of words. The advance in this knowledge has developed over the last 40 years with studies by Snowden et al.^30^ and Hodges et al.,^31^ mainly after the description of the slowly progressive aphasias by Mesulam in 1982.^32^

The temporopolar region is an important convergence site of multimodal association areas and paralimbic areas; this region sits at the "the downstream (deep) pole of the ‘what' pathway".^33^

The involvement of this area by strokes is relatively rare, and as our knowledge of the clinical-topographical relationships was largely based on studies of patients who suffered strokes (such as the cases described by Broca and Wernicke), it is possible that for this reason, the recognition of the importance of this region was so late. The renowned neurologist C. Miller Fisher stated that "we learn neurology stroke by stroke".^34^

The essential characteristic of semantic PPA is that, in addition to not being able to name an object or a living being, the individual also does not recognize the object or a living being when hearing its name.^30,31,33,35^ It is a loss of verbal meaning.

With increasing knowledge about the importance of the dominant hemisphere's temporopolar region for language, it became evident that the classical Wernicke-Lichtheim-Geschwind model of language neurobiology was incomplete.^36^

Figure 3B shows, in a simplified diagram, how these connections may be summarized today. According to the diagram, there is a dual language processing system where the phonological–motor aspects of speech are dependent on the dorsal regions of the brain, including the arcuate fascicle and Broca's area, whereas ventral regions support lexical-semantic processing.^36^

### Semantic processing

**Figure 5:**
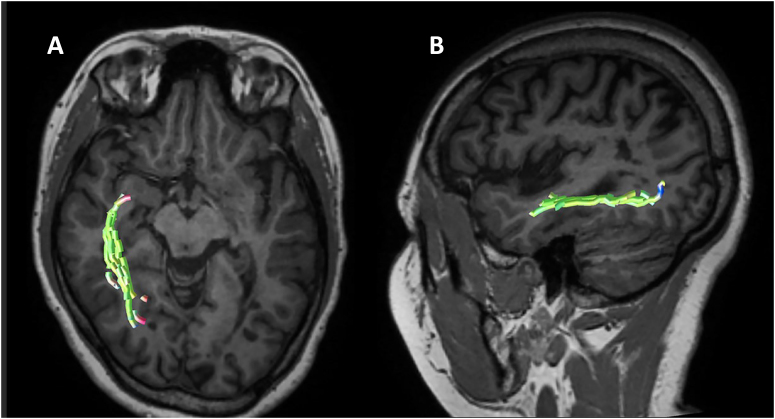
Inferior longitudinal fasciculus. Image A) Diffusion tensor magnetic resonance imaging; axial view; and B) Diffusion tensor magnetic resonance imaging; sagittal view. Courtesy of Diogo Fragoso Cardoso.

When semantic PPA began to be identified, there was an attempt to explain the semantic disorder based on the existence of a semantic center in the temporopolar region.^37^ But this idea was not reasonable, either from the neurophysiological or neuropsychological point of view, in which we understand that complex functions depend on complete functional systems^10-12,22^ or networks involving many connected areas.^16-18,23^ The most likely hypothesis was that when hearing a word or seeing the object or touching it, the temporopolar region is stimulated from the respective unimodal and multimodal association cortex. The connections through highly myelinated fascicles are essential for this process to be fast enough, with important participation of the inferior longitudinal fascicle (Figure 5) as well as the connections through the U-shaped fibers.^38^

There is no "dictionary" or a "semantic center" in the temporopolar region.^39^ This region probably plays a role similar to that of an amodal hub.^40,41^ As already mentioned above, when listening to the words of a text, broad but specific areas of the cerebral cortex are stimulated.

We tend to think that the central nervous system is always activated in a centripetal direction. According to this concept, stimuli from the external environment through vision, touch, hearing, and other senses activate the respective primary cortex and then the cortical areas of unimodal, multimodal, paralimbic association up to the regions of convergence such as the temporopolar region itself.^10,11,16,17^ This idea is correct but probably incomplete. It is known that these projections also occur in the opposite direction, allowing retroactivation.^16,17,42^ Retroactivation probably occurs from the hub located in the temporopolar region, which puts the semantic network into synchronic activity. An fMR study demonstrated that when a seed is placed in the temporopolar cortex, it is verified that there is an extensive network of areas in synchrony, which form the semantic network, showing that the anterior temporal lobe has intrinsic connectivity to an array of modality-selective primary and association cortices.^40^

In the semantic PPA, it is common to observe the occurrence of greater difficulty in naming living beings than man-made objects,^32,35,43^ which must mean earlier impairment of some neuronal groups than others in this region, simulating what occurs in lesions of specific cortical areas in which the individual presents anomy for colors or parts of the body. Likewise, it is possible to observe the progression from aphasia to visual agnosia for animals, objects, or people with the progression of the involvement of neuronal groups located in the temporopolar regions or their connections.^33,35,39,43^

Other conditions that also damage the temporal lobes, such as herpetic encephalitis, may rarely cause semantic aphasia, even with category-specific semantic disorders for biological categories.^44^

The development of the temporopolar region is very recent in phylogenesis. The temporopolar region is present only in primates and is much more developed in humans than in other primates.^33,45^ Furthermore, since Flechsig's studies at the beginning of the 20^th^ century, it is known that the myelination of the white matter of the occipitotemporal gyrus near the temporopolar regions, where there are afferent and efferent connections to and from the temporopolar region, is one of the most delayed in humans,^46^ preceding only the myelination of those to and from the anterolateral prefrontal cortex.

## SYNTAX: THE MOST IMPORTANT FEATURE OF HUMAN LANGUAGE

Another feature of human language that philosopher and linguist Noam Chomsky classifies as the most important and that best distinguishes it from the language observed in animals is the ability to string words together creating a new meaning or combining them into a sentence to convey meaning, an idea, or a proposal.^47^ The experiment carried out with the chimpanzee Nim Chimpsky, among those in which an attempt was made to verify whether an NHP could develop the ability to use syntax, was probably the one that gained the most notoriety.^48^ Nim Chimpsky was raised in a human family and trained to communicate through American Sign Language. Although controversies still persist, even regarding the development of a vocabulary and the interpretation of signs, there is an almost unanimous consensus that Nim Chimpsky did not develop the syntactic ability, even in a simple way.^48^

### Thought and language

In a recent review of the PPAs, Marsel Mesulam reported that the Russian psychologist Lev Vygotsky (1896–1934) compared thought to "a cloud shedding a shower of words".^49^

According to Chomsky, most of the time we use words to think and less time for communication.^47^ Vygotsky also paid much attention to the concept of "inner speech" and suggested that speech develops first in the social environment for communication, and later, becomes internalized into mental processes.^50^

### But, is it possible to think without language?

According to Piaget, during the sensorimotor stage (from birth to 2 years of age), children have a prelinguistic thought, in which action is the primary source of knowledge.^51^ Children grasp the concept of cause and consequence and object permanence before the development of language.^51^ NHPs also grasp the concept of object permanence,^52^ while the development of the concept of cause and consequence occurs in other animals, as shown by Ivan Pavlov (1849–1936) with studies of the conditioned reflex in dogs.^53^

The creation and use of tools by capuchin monkeys and gorillas indicate that thinking without language in animals is possible.^3,4^ Furthermore, thoughts that apparently seem to have less relationship with language are those related to feelings, that also occupy a lot of time in our mental activity.^54^ When we try to verbalize our feelings, we see how difficult this task may be. Try, for instance, to verbalize what you feel when thirsty or afraid. The ability to verbalize "our emotional experiences shape our lives in powerful ways".^55^ To verbalize our emotions may take many years of therapy before the emotions or feelings can be converted into internal language.

### Mirror neurons and language development

The discovery of mirror neurons by the group of Giacomo Rizzolatti et al., in 2004, had a great impact on explaining several behavioral and cognitive phenomena.^56^ Briefly, the discovery consisted of demonstrating that in monkeys, when motor neurons become active to carry out a propositional movement such as picking up an object, there are neurons in the frontal cortex near the motor area that become active in parallel, and those are called mirror neurons. And, most surprisingly, these mirror neurons also become active when another individual picks up this object.^56^ This phenomenon has been used to try to explain empathy and other behaviors,^57^ but it also helps to explain why primates can imitate the movements of other primates of the same or another species. Human homologs of monkeys mirror neurons were described.^58^

Spoken language development probably depended on imitation. Therefore, the emission of speech sounds by children, starting with lallation, occurs as an imitation of adult humans, and mirror neurons may have been important for this. But they are not enough. Other primates do not develop the capacity for verbal expression even when raised in a human family due to anatomic and physiological differences with the human brain.

It is noteworthy to mention here an observation by Charles Darwin: "The sounds uttered by birds offer in several respects the nearest analogy to language"^59^ — about the similarity between human language, with its long sentences, and the song of birds. Birds also learn to sing by imitation (they need auditory feedback) and learning only occurs until the age of sexual maturation.^60^ In addition, there is hemispheric lateralization.^60^ But there are no words, only singing.

It is interesting to observe how the sounds emitted by some monkeys, such as marmosets and capuchin monkeys, resemble birdsong, which makes us think that the analogy suggested by Darwin might be true. If these sounds emitted by capuchin monkeys are imitations of birdsong (listen to these sounds on our site – https://www.demneuropsy.com.br/wp-content/uploads/2024/01/DN-2023.0087-sound.m4a), that is, they are capable of imitating (or repeating) bird sounds, it is more likely that the inability to repeat phonemes and words depends more on the difficulty of expressing phonemes or words than on auditory identification or transmission from auditory areas to frontal areas of the brain.

### Genetics and language development

It is known that all human beings are born with the same ability to develop language, which means that the genetic changes that allowed human language occurred before the great emigration of *Homo sapiens* from Africa to other regions of the world, which occurred about 50 to 60 thousand years ago.^47^

It is possible that these mutations occurred sequentially or that only one mutation allowed the development of language. Based on the existence of rudimentary forms of language in NHPs, the hypothesis of many mutations in sequence seems more likely.

A finding that apparently contradicted the hypothesis that many mutations would be necessary to allow the development of language ability occurred when, in the study of the KE family, in which many members had great language difficulty, both in expressing and understanding, a mutation was discovered on the FOXP2 gene, located on chromosome 7.^61^

This gene is responsible for the synthesis of the forkhead box P2 protein that controls the activity of many genes. Thus, this protein is involved in many processes, including neuron development and neurotransmission. The forkhead box P2 protein appears to be essential for the normal development of speech and language.^62^

The discovery of differences in two amino acids between the forkhead box P2 proteins of humans and chimpanzees raised the possibility that the mutation in FOXP2 gene could be one of the main factors for the development of human language.^61-63^

But soon after, analysis of Neandertal DNA verified that our closest relative already shared with modern humans these two evolutionary changes in FOXP2.^64^ The result suggested that the two amino-acid substitutions had occurred 400 thousand years ago or even before in a common ancestor of modern humans and Neandertals.^63,64^

This relevant finding, once excluded the possibility of inbreeding, showed that language (if normal human FOXP2 gene is the precondition for language development) could be present in other members of the genus *Homo*. There are controversies about the possibility that Neandertals shared with us something like modern language.^47,65,66^ However, although genetics is a promising window into the neurobiology of speech and language, we should not expect the neurobiology of language to be explained by a single gene.^65^

In conclusion, there are several differences between humans and NHPs but the most important is the development of language in humans. Brain areas and networks related to language are much more developed in humans than in NHPs.

NHPs have rudimentary forms of language used for communication of events in real-time, but their lexical-semantic system is very poor with a "vocabulary" of only a few words while humans may have a dictionary in their brain with 20,000 to 35,000 words.^26^ Language was probably due to imitation behavior. Above and beyond this huge vocabulary, humans developed syntax, which increases the complexity and power of language. Language started for communication but later it was internalized and became more related to thinking than communication. With this empowerment of thinking, due in great part to the development of grammar, language interferes with all higher cortical functions.
